# Modification Mechanism of Low-Dosage Vinyl Acetate-Ethylene on Ordinary Portland Cement–Sulfoaluminate Cement Binary Blended Rapid Repair Mortar

**DOI:** 10.3390/polym17111501

**Published:** 2025-05-28

**Authors:** Hecong Wang, Yuxue Zhu, Ting Li, Xiaoning Li, Shuai Peng, Jinzhu Guo, Xuqiang Pei, Congchun Zhong, Yihang Yang, Qiang Ma, Zhonglun Zhang, Minghui Wu, Qunchao Zhang, De’an Shi, Zuobao Song

**Affiliations:** 1 CNBM Zhongyan Technology Co., Ltd., Beijing 100024, China; wanghecong@cbma.com.cn (H.W.); zhuyuxue@cbma.com.cn (Y.Z.); liting@cbma.com.cn (T.L.); lixiaoning@cbma.com.cn (X.L.); pengshuai@cbma.com.cn (S.P.); guojinzhu@cbma.com.cn (J.G.); peixuqiang@cbma.com.cn (X.P.); zhongcongchun@cbma.com.cn (C.Z.); yangyihang@cbma.com.cn (Y.Y.); maqiang@cbma.com.cn (Q.M.); zhangzhonglun@cbma.com.cn (Z.Z.); wuminghui@cbma.com.cn (M.W.); 2 China Building Materials Academy, Beijing 100024, China; 3 School of Materials Science and Engineering, Hubei University, Wuhan 430062, China; zhangqc1976@hubu.edu.cn (Q.Z.); deanshi2012@hubu.edu.cn (D.S.)

**Keywords:** polymer-modified mortar, VAE, mechanical properties, microstructure, rapid repair

## Abstract

This study developed a vinyl acetate-ethylene rapid repair mortar (VAE-RRM) by using a binary blended cementitious system (ordinary Portland cement and sulfoaluminate cement) and vinyl acetate-ethylene (VAE) redispersible polymer powder. The effects of the polymer-to-cement ratio (P/C: 0~2.0%) on setting time, mechanical properties, interfacial bonding, and microstructure were systematically investigated. The results reveal that VAE delayed cement hydration via physical encapsulation and chemical chelation, extending the initial setting time to 182 min at P/C = 2.0%. At the optimal P/C = 0.9%, a synergistic organic–inorganic network enhanced flexural strength (14.62 MPa at 28 d, 34.0% increase) and interfacial bonding (2.74 MPa after interface treatment), though compressive strength decreased to 65.7 MPa due to hydration inhibition. Excessive VAE (P/C ≥ 1.5%) suppressed AFt/C-S-H growth, increasing harmful pores (>1 μm) and degrading performance. Microstructural analysis via scanning electron microscopy (SEM) and mercury intrusion porosimetry (MIP) demonstrates that VAE films bridged hydration products, filled interfacial transition zones (ITZ), and refined pore structures, reducing the most probable pore size from 62.8 nm (reference) to 23.5 nm. VAE-RRM 3 (P/C = 0.9%) exhibited rapid hardening (initial setting time: 75 min), high substrate recovery (83.3%), and low porosity (<10%), offering an efficient solution for urban infrastructure repair. This work elucidates the dual mechanisms of pore refinement and interface reinforcement driven by VAE, providing theoretical guidance for designing high-performance repair materials.

## 1. Introduction

As an important part of the new urbanization strategy, urban renewal has become an important issue in the field of urban planning. With the rapid progress of urbanization in China and the continuous growth of infrastructure service time, some old buildings are in disrepair, and the main body of the building is damaged to different degrees, such as cracks, falling blocks, and structural strength decline damage. These structural performance degradation problems not only affect the use of building functions but also may cause major safety hazards. Based on this, many old buildings in the city can no longer meet the needs of modern life and are in urgent need of restoration and renewal. With the in-depth promotion of the concept of sustainable development, engineering restoration is facing more stringent technical standards and audits in terms of timeliness, quality, reliability, and environmental friendliness. Traditional repair materials have poor adhesion, insufficient durability, low early strength, and other inherent defects, making it difficult to meet the real needs of quick repair and long-term use. In this context, polymer-reinforced mortar (PRM) came into being, which synthesizes the existing advantages of polymer materials and inorganic materials [1,2,3,4], providing a new way to solve existing repair problems and enhance the efficiency of urban renewal.

PRM is a traditional cement mortar with a polymer component that improves its performance. Traditional cement mortar has high strength [5,6,7] but also significant deficiencies in properties such as flexural strength and modulus of elasticity [8,9,10]. The incorporation of polymer additives in cement mortar facilitates the formation of an interconnected three-dimensional network structure within the matrix. This polymeric network effectively encapsulates cement particles and hydration products while enhancing the interfacial bonding between aggregates and cementitious phases. [11,12,13,14], and enhancing the mechanical and durability properties of the material [15].

At present, for the repair of polymer-reinforced mortar, experts have conducted a lot of research, with the common polymers used in PRM mainly being vinyl acetate-ethylene (VAE) [16], acrylic (AC) [17], styrene-acrylic emulsion (SAE) [18], and styrene-butadiene rubber (SBR) [19,20]. Pei et al. [17]. studied the effect of acrylic emulsion dosage on the setting time, mechanical properties, bonding properties, and shrinkage properties of cement-reinforced mortar and analyzed it from a microscopic perspective. Their study found that the increase in emulsion would prolong the setting time of mortar, reduce the shrinkage rate, and improve volume stability. When the acrylic emulsion dosage was 16.2%, the 28 d shrinkage rate could reach 0.08%. Shi et al. [21] investigated the effects of VAE and methyl cellulose on the performance of PRM. They found that the synergistic action of these two components significantly improves flexural strength: the addition of VAE promotes the formation of ettringite (AFt), while methyl cellulose ensures more uniform distribution of hydration products to avoid strength inversion. Yu et al. [15] explored the mechanism of impermeability enhancement in reinforced mortar using scanning electron microscopy (SEM), in situ nondestructive testing, and other methods. Through microscopic analysis, they concluded that the polymer film formed on the surfaces of cement and aggregates fills the pores between hydration products, thereby improving the impermeability of the reinforced mortar.

According to the selection of different cementitious materials, the materials can be divided into ordinary Portland cement polymer-reinforced mortar (OPCPRM) [22], sulfoaluminate cement polymer-reinforced mortar (SACPRM) [23], and polymer repair and reinforcement mortar with a variety of cementitious materials in the compounding system (CPRM) [24]. Sun et al. [24] investigated the strength and durability of CPRM and found that by using a composite cementitious material system, the setting time can be shortened and the overall partial durability can be improved. Compared with a single cementitious material system, it has better performance.

At present, most of the academic research [25,26] focuses on the polymer component in the proportion of cement mixing between 5% and 15%, although the polymer added to the reinforced mortar can play a very good role in improving the working performance and flexural and adhesive properties, but through our preliminary experiments, we found that when the VAE content exceeds 2%, it significantly reduces the compressive strength and prolong the setting time. While polymer-modified cement mortar demonstrates enhanced performance through optimized polymer-cement interactions, its practical implementation in engineering applications necessitates further systematic investigation.

This paper aims to study the effect of low VAE powder doping on OPC and SAC binary compound polymer-reinforced mortar for the current demand of building reinforcement repair. By analyzing the working and mechanical properties of mortar under different VAE dosage conditions and the influence of different interfacial treatments on its adhesive properties, combining SEM, EDS, MIP, and other analytical means, the influence mechanism of VAE powder on the comprehensive performance of polymer reinforced mortar is revealed.

## 2. Materials and Methods

### 2.1. Materials

Cementitious material: mixed cementitious material, compounded from 42.5 ordinary Portland cement (OPC) and sulfoaluminate cement (SAC) according to a certain ratio, industrial grade; CNBM Zhongyan Technology Co., Ltd., Beijing, China. The physical performance indexes and chemical composition of the cementitious materials used in specific tests are shown in Table 1 and Table 2 below:

Sand: ISO standard sand for experiments, Xiamen ISO standard sand CO., Ltd., Xiamen, China.

**Figure 1:**
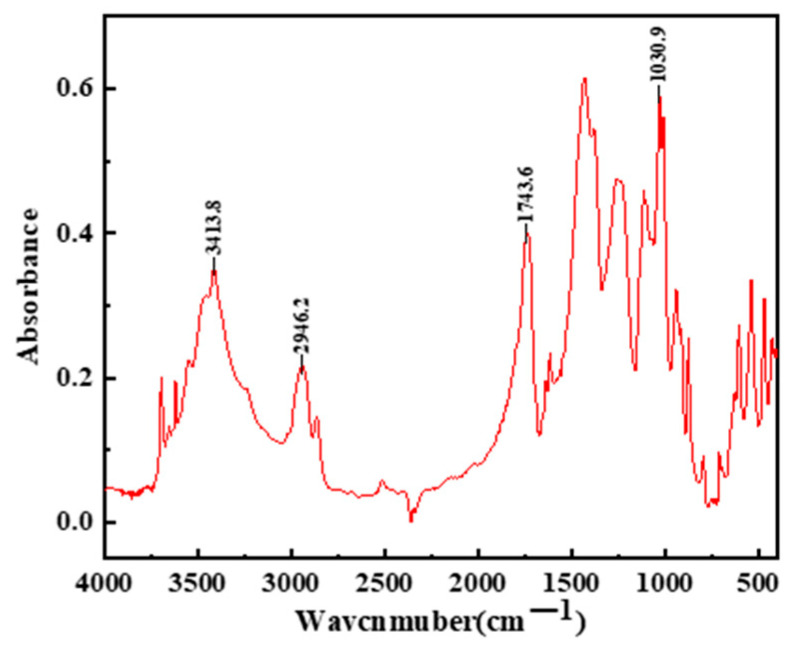
FTIR spectrum of VAE powder.

Dispersible latex powder: A vinyl acetate-ethylene (VAE) copolymer powder (Grade 5010, Wacker Chemicals (China) Co., Ltd., Shanghai, China.) was used in this study; the basic properties of the VAE powder are shown in Table 3. Fourier-transform infrared spectroscopy (FTIR) analysis (Figure 1) confirmed the presence of reactive functional groups, including carboxyl (-COOH) and ester (-COOR) linkages within the polymer structure.

Interface treatment agent: A VAE emulsion-type interface treatment agent. Industrial grade; CNBM Zhongyan Technology Co., Ltd, Beijing, China..

### 2.2. Sample Preparation

This experiment aimed to study the effect of different adhesive powder dosages on the performance of reinforcement repair mortar, with the P/C in the range of 0–2%. Water–cement ratio, W/C; cement-to-sand ratio, C/S; water-reducing agent dosage in the cement dosage of the mass ratio of C/CR.

The VAE-RRM base ratio for C:W:S:CR = 1:0.32:1:0.05; the specific ratio is shown in Table 4.

### 2.3. Test Instruments

Cement sand mixer: Model JJ-5, Wuxi Jianyi instrument & nachinery Co., Ltd., Wuxi, China; Electronic balance: Model JJ1000A, G&G Measurement Plant. Changshu, China; Mortar flexural and compressive machines: Model DKZ-6000, Wuxi Jianyi instrument & nachinery Co., Ltd. Wuxi, China; Model Instron 5, Shenzhen Wance Testing Equipment Co., Ltd. Shenzhen, China; JG-259 Intelligent Bond Strength Tester: Model HC-2000A, Beijing High-chance High-tech Science Co., Ltd. Beijing, China; Environmental scanning electron microscope(ESEM): QUANTA 250G, FEI Company, Hillsboro, OR, USA. Mercury Pressure Meter(MIP), AutoPore IV 9510, Mcmurrittick (Shanghai) Instrument Co., Ltd. Shanghai, China.

### 2.4. Test Experiments and Characterization Methods

#### 2.4.1. Setting Time Test

We refer to DL/T 5126-2001 [27] Test code on polymer-modified cement mortar” [25]. During the test, the mixed net cement mortar was put into the test mold, and the initial and final setting time of the polymer-reinforced repair mortar was determined using Vickers apparatus. The setting time was determined through two repeated experiments, and the mean value was adopted as the final result. If the discrepancy exceeded the permissible range, additional tests were conducted.

#### 2.4.2. Mechanical Performance Test

With reference to GB 50728-2011 “Technical code for safety appraisal of engineering structural strengthening materials” [28] on the reinforcement mortar force for academic performance testing, we used a test block molding size of 40 mm × 40 mm × 160 mm. Each ratio was molded in a set of triple molds for testing, and after the slurry was poured into the mold, it was wet-conserved for 3 d. Then, we removed the side mold of the sample block, kept the bottom mold, and continued with wet conservation for 25 d until testing.

Flexural strength test: The cured mortar specimens were placed on the flexural testing machine with the molded side facing upward. A three-point bending configuration was adopted, aligning the specimen’s longitudinal axis perpendicular to the support rollers. Loading was applied at a constant rate of 50 ± 10 N/s using a closed-loop control system. During loading, care was taken to maintain uniform force distribution and prevent specimen tilting. The test was terminated upon visible fracture initiation, and the peak load at failure was recorded. Each mix ratio was tested independently. The flexural strength was selected from the average value of three specimens. If the maximum or minimum value differed from the average value by more than 15%, additional specimens were included for re-testing.

Following the flexural test, the two broken prism segments were immediately subjected to compressive testing. Each segment was positioned in a 40 mm × 40 mm steel platen fixture, ensuring centering of the prismatic cross-section. Axial compression was applied at a rate of 2400 ± 200 N/s until structural failure occurred. The maximum load sustained prior to collapse was documented for strength calculation. Each mix ratio was tested independently. The compressive strength was selected from the average value of three specimens. If the maximum or minimum value differed from the average value by more than 15%, additional specimens were included for re-testing.

Compression–flexure ratio: The same specimen in the flexural and compressive strength test was used. The resulting ratio is the result of the compression and folding ratio, which can be used to assess the performance of the mortar. If the mortar compression and folding ratio is small, this indicates that the mortar can maintain better stability and resistance to deformation when it is subjected to external forces.

The compression–flexure ratio is calculated using Equation (1):

(1) $$T=\frac{R_{c}}{R_{f}}\times 100\%$$

where:

T: compression–flexure ratio;

$R_{c}$: flexural strength (MPa);

$R_{f}$: compressive strength (MPa).

#### 2.4.3. Bonding Strength Test of Different Substrate Treatments

We conducted bonding strength tests with reference to GB 50728-2011 for the reinforced mortar. In order to fully simulate the field environment, the base cement mortar block had a dry base surface, a wet base surface, and an interfacial agent-coated base surface that had undergone three kinds of processing. To ensure that the bonding surface was dry, we dried the substrate test block with a wire brush to remove surface and floating dust. The wet substrate test block was dried after being soaked in water for 1 d, and the surface water shall be wiped off before the bonding test; The coating treatment of interface agent shall be evenly applied on the dry test block, and the amount of interface agent shall be 0.5 kg/m^2^. The calculation results need to remove the maximum and minimum values of the test sample values, and calculate the average value of the remaining three samples as the bonding strength.

#### 2.4.4. Interfacial Flexural–Tensile Strength Test

**Figure 2:**
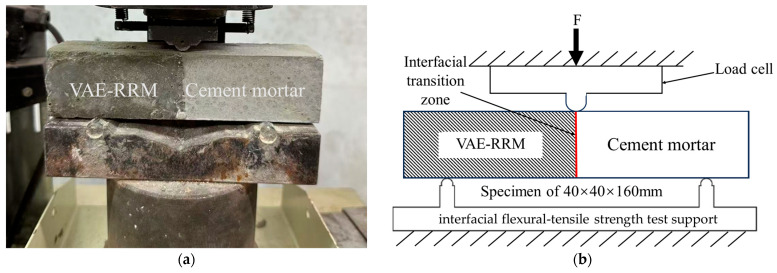
Interfacial flexural–tensile strength test of cement mortar. (**a**) Experimental set-up; (**b**) diagrammatic sketch.

According to the interfacial flexural–tensile strength test method specified in JC/T 2381-2016 “Repairing mortar” [29] shown in Figure 2, we used a standard sand/water ratio = 1:3:0.5 and a mold size of 40 mm × 40 mm × 80 mm as the matrix specimen. After 28 d of maintenance under standard conditions, the matrix specimen was put into the 40 mm × 40 mm × 160 mm triple mold. The polymer-reinforced mortar was cast into the other half of the mold and was kept under standard conditions of maintenance for 28 d to test its flexural strength. Each group contained six specimens. The calculation results need to remove the maximum and minimum values of the test sample values, and calculate the average value of the remaining four samples as interfacial flexural-tensile strength.

#### 2.4.5. Mercury Intrusion Porosimetry (MIP) Test

Mercury has non-wetting properties for most solid materials and requires external pressure to enter solid pores. For cylindrical pore models, the size and pressure of the pores that mercury can enter follow the Washburn equation. By controlling different pressures, the volume of mercury injected into the pores can be measured, and the cumulative distribution curve or differential curve of the pore size corresponding to different pressures can be obtained. The analytical aperture range is 5 nm to 800 µm, and 2 g samples for each mix ratio were needed to conduct the MIP test.

#### 2.4.6. Microcosmic Observation Experiment

The microstructure of the hydration products of polymer-reinforced mortar with different dosages of adhesive powder was observed through an SEM experiment. The distribution of the polymer adhesive powder was observed, the influence mode was determined, and the composition of the observed area was analyzed by EDS, with the detection range of the spectrometer being B5-U92. By analyzing the microstructure, the specific function mechanism of the VAE adhesive powder in the polymer-reinforced mortar was investigated, and the optimal proportioning scheme was determined on this basis.

**Figure 3:**
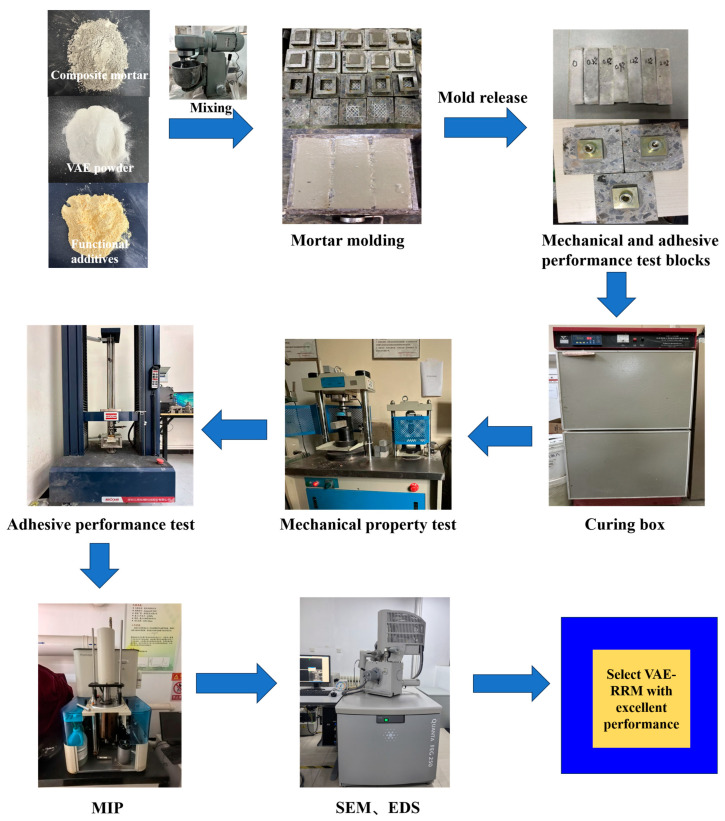
A flowchart of the experiment methodology.

The specific experimental steps are shown in Figure 3 below:

## 3. Results and Discussion

### 3.1. Results

#### 3.1.1. Setting Time

**Figure 4:**
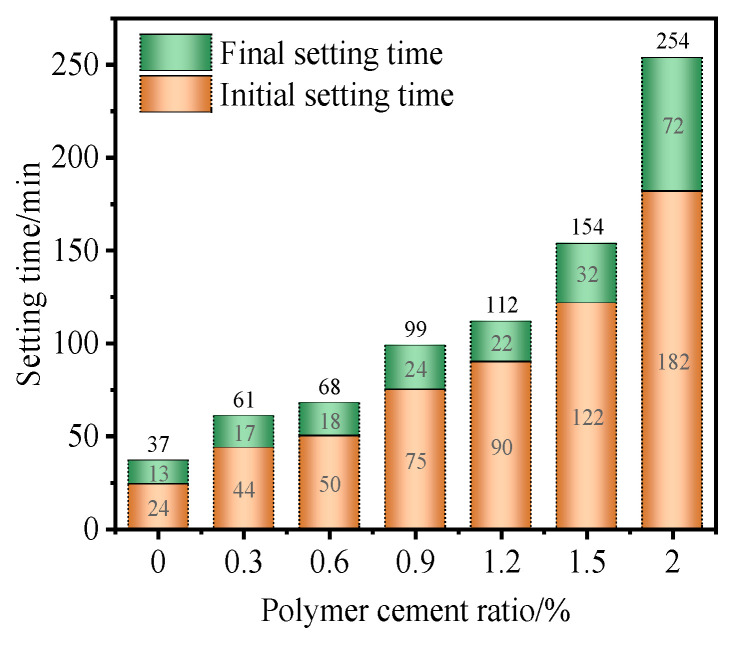
Setting time under different polymer-to-cement ratios.

The setting time of the polymer-reinforced mortar was prolonged with the increase in the dosage of glue powder, which had a certain impact on the operational performance of the reinforced repair mortar as well as the construction time. Through the experimental tests, it was found that an appropriate amount of adhesive powder can ensure the strength of the mortar whilst maintaining its working performance. The specific test data are as follows:

As can be seen from Figure 4, the cementitious material is a ordinary Portland cement (OPC) and sulfoaluminate cement (SAC) compound system. The composite system contained Portland cement as the main cementitious material. After adding alumina sulfate cement, C_4_A_3_S and gypsum rapidly contacted each other to generate a large number of AFt and release a large amount of hydration heat. The generated AFt also served as a nucleation matrix for the hydration products of C_3_S and C_2_S in the OPC, which further accelerated the hydration of silicate minerals and thus achieved the effect of quick setting and early strength [30]. In the absence of VAE powder, the benchmark group of the initial setting time was 24 min, and the final setting time was 37 min.

With the increase in the doping amount of adhesive powder, the solidification process of the reinforced repair mortar was affected. Specifically, an increase in the dosage of rubber powder led to a delay in the mortar’s setting time, and the extent of this delay became more significant. When the P/C reached 0.9%, the initial setting time of the polymer-reinforced mortar was 75 min, and the final setting time was 99 min; compared with the cement mortar, the initial and final setting time prolongation reached 215.5% and 167.6%. When the P/C was increased from 1.5% to 2.0%, the initial and final setting time of the repair mortar were significantly prolonged to 182 min and 254 min.

Upon analyzing this trend, it is thought that the VAE particles added in the mortar formed a film-like structure upon contact with water and attached to the surface of the cement particles, affecting the contact area of the cement particles with water and thus affecting the hydration process of cement. At the same time, the carboxyl group in the VAE had a complex reaction with the Ca^2+^ ions in the mortar, reducing the concentration in the solution, delaying the formation of hydration products, and thus prolonging the setting time; on the other hand, the formation of the membrane-like structure of the gum powder generated chemical adsorption on the surface of cement, resulting in spatial site resistance between cement particles and thus affecting particle cohesion. In conclusion, the addition of VAE affected the setting time of VAE-RRM through both chemical and physical effects.

#### 3.1.2. Flexural Strength

**Figure 5:**
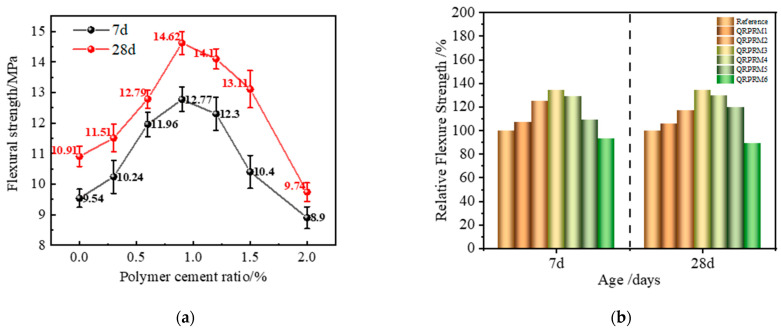
The flexural strength under different polymer-to-cement ratios. (**a**) The flexural strength of VAE-RRM; (**b**) the relative flexural strength of VAE-RRM.

Flexural strength is an important index for measuring the performance of mortar. It can react to the resistance of mortar during bending or breaking. For polymer-reinforced mortar, high flexural strength can ensure that even when subjected to long-term cyclic loading or environmental erosion, it can still maintain its performance to meet the needs of the project. The flexural strength of reinforced mortar under different polymer–ash ratios was investigated, and the results are as follows:

Observing the flexural strength test results in Figure 5, it can be seen that along with the addition of VAE adhesive powder, the overall flexural strength of the reinforced repair mortar showed a trend of increasing and then decreasing, with the P/C in the range of 0–0.9%. The maximum value occurred when the P/C reached 0.9%, with the 7 d flexural strength being 12.77 MPa and the 28 d flexural strength being 14.62 MPa. Compared with the benchmark group (7 d 9.54 MPa, 28 d 10.91 MPa), the flexural strength increased by 33.9% and 34.0%; when the P/C was increased to 1.5%, the flexural strength decreased, but it was still higher than that of the benchmark group. Analyzing the above data, it can be concluded that the addition of gum powder has an enhancing effect on the flexural performance of mortar, and the addition of an appropriate amount of gum powder can cause a significant increase in the flexural strength.

Analysis of the reason shows that the addition of VAE powder can enhance the flexural strength of mortar, mainly because before the addition of VAE powder, the internal mortar was mainly connected with the hydration products of cement to build a kind of cement mortar mesh structure. Upon the incorporation of glue powder, its particles filled the pore spaces within the mortar and formed emulsion films, which densified the internal structure of the mortar, optimized the pore structure of the mortar matrix, and enhanced the compactness of the interfacial transition zone (ITZ). This dual effect of pore structure optimization and ITZ compactness improvement resulted in a more homogeneous and dense internal system of the cement mortar. On the other hand, the latex film was wrapped and attached to the hydration products of cement, which can play the role of overlap, so that the mortar was able to form a kind of organic–inorganic mesh structure from the inorganic mesh structure. When subjected to external forces, The rigid cement hydration product structure will no longer bear the load alone, but the introduced polymer component and cement hydration product component together form a composite material to bear the load, so as to improve the flexural strength., However, when the VAE powder amount continued to increase, this resulted in an increase in the proportion of organic components in the reticulation structure occupying the position of the hydration products such as Aft and C-S-H gel. The organic component occupied the composite reticulation structure to a greater degree, but the bearing capacity of polymer membrane is far lower than that of cement hydration products, resulting in the reduction of flexural strength.

#### 3.1.3. Compressive Strength

**Figure 6:**
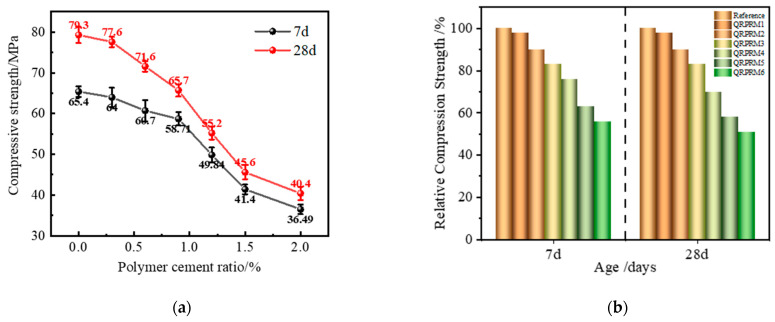
Compressive strength under different polymer-to-cement ratios. (**a**) The compressive strength of VAE-RRM. (**b**) The relative compressive strength of VAE-RRM.

Compressive strength is related to the bearing capacity of mortar under load. As a reinforcing and repairing material, the mortar’s strength index needs to meet the stability and safety requirements of the building structure. We tested the compressive strength of different P/C values, and the results are as follows:

As shown in Figure 6, the compressive strength generally showed a decreasing trend with the increase in VAE powder doping. When the experimental group doped with gum powder was compared with the benchmark group, there was a significant decrease in the compressive strength, especially when the P/C was increased from 0.9% to 1.5%, and this decreasing trend was the most significant. At 7 d, the compressive strength of the benchmark group was 65.4 MPa, and the compressive strength under P/C 0.9% and 1.2% decreased to 58.7 MPa (89.8%) and 49.8 MPa (76.2%), respectively; the compressive strength was increased at 28 d, but the addition of the VAE adhesive powder had an unfavorable effect on increasing the compressive strength. The compressive strength under P/C 0.9% and 1.2% decreased to 58.7 MPa (89.8%) and 49.8 MPa (76.2%), respectively. Under P/C 1.2%, the compressive strength was 65.7 MPa (82.8%) and 55.2 MPa (69.6%), with the relative compressive strength decreasing by 7.0% and 6.6%, respectively. When the P/C increased to 1.5~2.0%, it had a greater impact on the polymer-reinforced mortar, with the relative compressive strength dropping to less than 60%, resulting in the compressive strength not complying with the requirements of GB 50728-2011 reinforcement material standards.

After adding glue powder to the mortar, its small polymer particles emulsified with water to form a latex film structure that adhered to the surface of cement particles, affecting the dissolution of active minerals in cement and the nucleation products and positions of cement hydration, thereby hindering the process of cement hydration; as the amount of glue powder increased, more cement particles were affected, and the conclusion is consistent with the data results of the 7 d compressive strength. As the curing age increased, the moisture in the latex film wrapped on the cement particles continued to react with the cement particles and generate hydration products, thereby causing the compressive strength to continue to increase. However, the polymer film covered the surface of the hydration products, affecting their formation and growth and resulting in a low degree of compressive strength growth, which impacted the final compressive strength.

#### 3.1.4. Compression–Flexure Ratio

**Figure 7:**
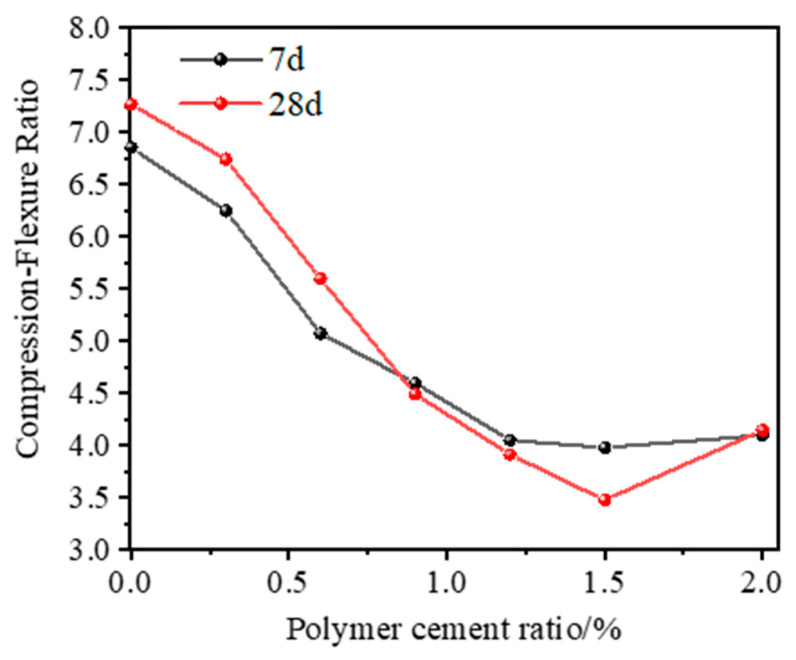
Compression–flexure ratio under different polymer-to-cement ratios.

The compression–flexure ratio can be used to evaluate the performance of mortar. The smaller the compression–flexure ratio of mortar, the better the stability and deformation resistance when subjected to external force, and the better the toughness. Figure 7 shows the compression–flexure ratio under different P/C values. As the P/C increases, the compression–flexure ratio of mortar decreases significantly, indicating that the addition of polymer powder has a toughening effect on mortar. As shown in Table 5, when the P/C is 0.9%, the 28 d compression–flexure ratio is 4.49, which is 50.1% lower than the baseline group; although the compression–flexure ratio still decreases from 1.2% to 2.0%, the actual decrease in the mortar flexural strength is less than that in the compressive strength, so the compression–flexure ratio value is further reduced. Based on the above data, it is believed that the comprehensive mechanical properties are optimal when the P/C is 0.9%.

#### 3.1.5. Bonding Performance of Different Interface Treatment Methods

**Figure 8:**
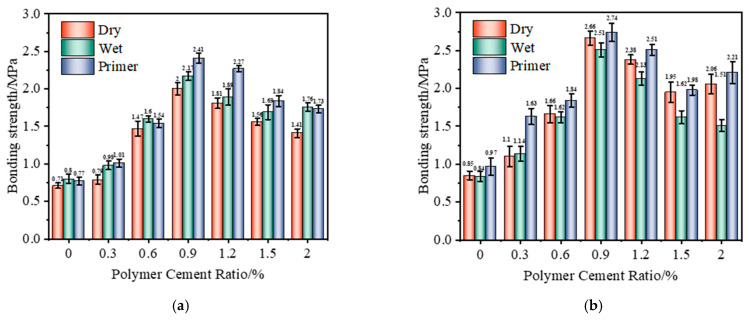
Bonding strength under different polymer-to-cement ratios: (**a**) 7 d; (**b**) 28 d.

The bonding performance of reinforcement and repair mortar is a very important aspect of building structure. Good bonding performance can ensure a strong bond between mortar and various substrates, which is crucial to prevent peeling between materials and improve the bearing capacity of the overall structure. The bonding performance under different P/C values was tested, and the specific results are as follows:

As shown in Figure 8, with the increase in the amount of VAE powder, the bonding strength of different base surface treatments at 7 d and 28 d showed a trend of first increasing and then decreasing, among which the interface agent coating treatment had the best effect and the dry treatment had the worst effect. When the P/C reached 0.9%, the bonding strength reached the maximum value, and the bonding strength of the 7 d dry base surface, wet base surface, and emulsion coating base surface reached 2.00 MPa, 2.41 MPa, and 2.51 MPa, respectively; compared with the 7 d benchmark group, the bonding strength increased by 181.7%, 213.0%, and 198.8%, respectively. The bonding strength of the 28 d dry base surface, wet base surface, and emulsion coating base surface reached 2.17 MPa, 2.66 MPa, and 2.74 MPa, respectively; compared with the 28 d benchmark group, the bonding strength increased by 171.3%, 213.0%, and 182.5%, respectively. Analysis of the reasons shows that the emulsion interface agent contains a large number of organic molecules which can fill the mortar pores and capillaries of the old base layer, optimize the internal pore structure of the mortar, and make the material more compact; at the same time, the interface agent treatment can make the contact layer between the mortar and the old base layer have good wettability and can better bridge the cement matrix and aggregate of the new and old mortars [31].

#### 3.1.6. Interfacial Flexural–Tensile Strength Test Results

Before the interfacial flexural–tensile strength test, the 28 d curing foundation test blocks were first selected for bending tests to determine the bending strength of the foundation test blocks. As shown in Table 6, the bending strength of the foundation test blocks to be repaired at 7 d and 28 d was 10.1 MPa and 10.3 MPa, respectively, so the bending strength of the foundation test blocks was stipulated to be 10.2 MPa.

**Figure 9:**
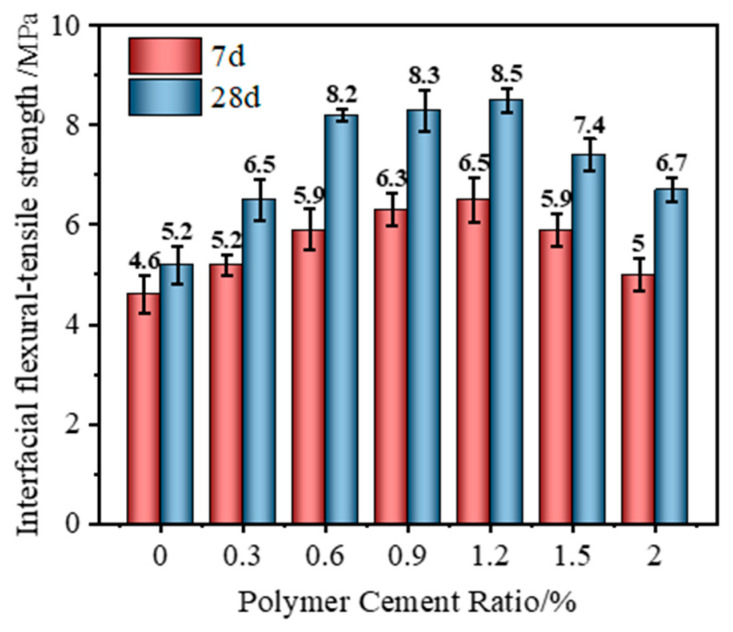
Interfacial flexural–tensile strength of VAE-RRM with different polymer-to-cement ratios.

As shown in Figure 9, with the increase in the P/C, the interfacial flexural–tensile strength showed a trend of first increasing and then decreasing. After 7 d of curing un-der standard conditions, the interfacial flexural–tensile strength could recover to about 45~60% of the matrix strength and reached the highest when the P/C was 1.2%, reaching 63.7% of the matrix strength and 52.8% of the strength of the repair material itself. The 28 d interfacial flexural–tensile strength could recover to about 65–80% of the matrix strength and reached the highest when the P/C was 1.2%, reaching 83.3% of the matrix strength and 60.3% of the strength of the repair material itself.

#### 3.1.7. Mercury Intrusion Porosimetry (MIP)

The pores in polymer-reinforced mortar can be divided into four categories: gel pores (<10 nm), transition pores (10–100 nm), capillary pores (100–1000 nm), and macropores (>1000 nm). Among them, gel pores will have a certain impact on the shrinkage properties of the mortar; transition pores and capillary pores will affect the permeability and partial strength of the mortar; and macropores will mainly affect the mechanical properties of the mortar.

**Figure 10:**
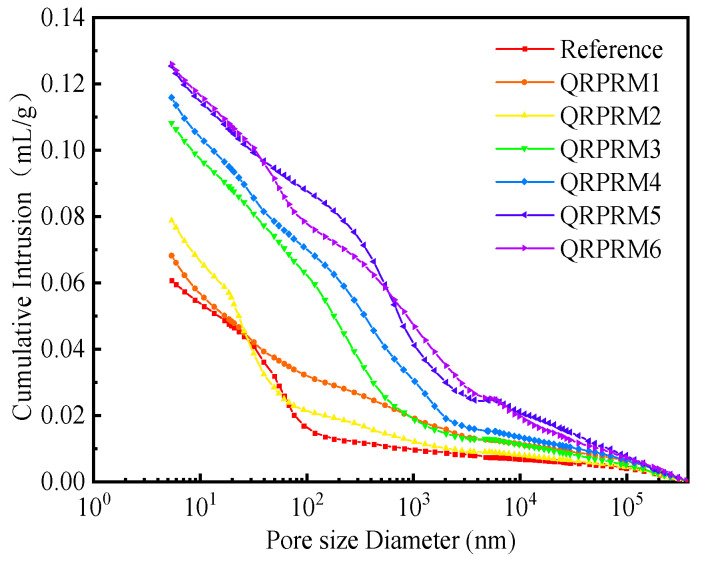
Influence of VAE ratio on cumulative pore volume of VAE-RRM.

It can be seen from Figure 10 that with the increase in the P/C, the total porosity of VAE-RRM gradually increased. This is mainly due to the air entrainment effect caused by the addition of VAE, which caused the polymer film to remain inside the mortar during the cement hydration process, thereby affecting the strength. This is consistent with the mechanical property results obtained in this study.

**Figure 11:**
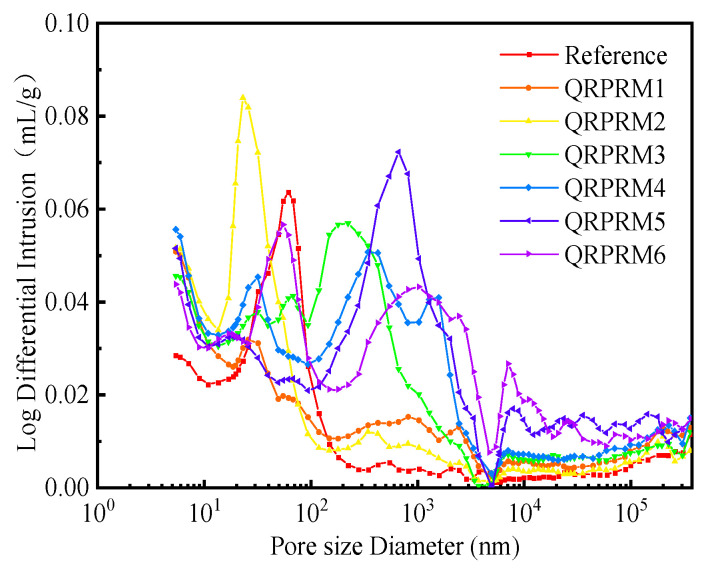
Log differential mercury input of VAE-RRM.

Figure 11 shows the distribution of VAE-RRM pore volume under different P/C values. With the increase in the P/C, the most probable aperture size shows a trend of decreasing and then increasing; in the range of 0–0.6%, the most probable aperture size is mainly distributed in the range of 10–100 nm, whereas the most probable aperture size of the benchmark group reaches 62.8 nm, and the pore size decreases to 23.5 nm when the P/C reaches 0.6%, which is 57.1% smaller than that of the benchmark group. The reason is that the addition of VAE closes and fills the harmful holes in the cement paste, separates the large harmful pores from the polymer film into smaller transition pores, and reduces the number of large holes and capillaries, and the most probable pore size can migrate towards smaller transition pores, resulting in a denser mortar structure, which makes the structure of the mortar more dense and improves the flexural performance and adhesive properties of the VAE-RRM. When the P/C is 0.9%, the most probable aperture sizes become large pores, but the overall pore size distribution shows a trend of multi-peak distribution. Compared with other ratios of reinforced mortar, VAE-RRM 3 has a pore size in the range of capillary and transition holes dispersed more uniformly and continuously, and the peak area is concentrated in the range of 30 nm to 220 nm, which indicates that the addition of VAE adhesive powder can optimize the pore structure, making the distribution of pores more uniformly dense; thus, the mechanical properties and adhesive properties have better development [32,33].

When the P/C continues to grow, the size of the most probable aperture sizethethe increases. The pore distribution changes from a single-peak distribution to a bimodal distribution, and the main peak size better resembles capillary pores. On the one hand, the increase in the dosage of VAE in the mortar as a whole exerts an air-entraining effect; on the other hand, the loss of water in the glue powder film results in the formation of pores. With overdoping of VAE, the hydration of the cement is significantly affected, resulting in the deterioration of the internal structure. The size of the shoulder peak gradually decreases as the P/C increase, and the pore size is smaller than the most probable aperture size of the benchmark group indicating that VAE still plays a role in optimizing pore structure. However, the deterioration brought about via the overdoping of VAE has a greater impact, which leads to a significant reduction in the mechanical properties and adhesive properties of VAE-RRM.

**Figure 12:**
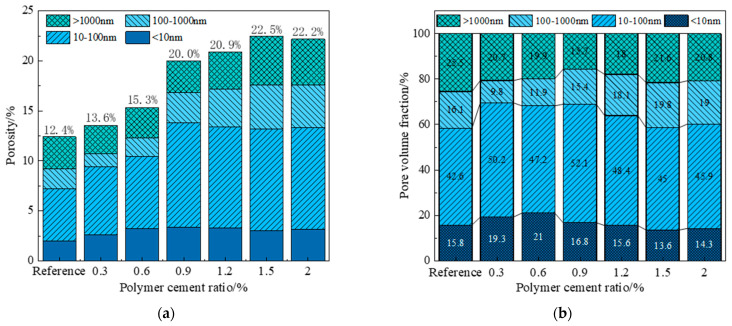
Influence of VAE on pore size distribution of VAE-RRM: (**a**) total porosity; (**b**) proportion of various aperture types.

Figure 12 shows the pore size distribution under different P/C values, and it is found that with the increase in VAE doping, the proportion of gel holes and transition holes shows a trend of increasing and then decreasing, and the proportion of gel holes and transition holes reaches 68.9% when the P/C is 0.9%, which improves by 10.5% compared with that of the benchmark group, showing that within a certain range of doping, VAE is able to reduce the generation of large pores. The proportion of transition holes and capillaries increases significantly, which may be attributed to the addition of the polymer to form a polymer membrane structure, eliminating some of the harmful pores and then converting them into smaller pores, which has a positive effect on the mortar structure as a whole, the same as the conclusion obtained from the above analysis.

#### 3.1.8. Scanning Electron Microscope Micrograph (SEM) of VAE-Modified Reinforced Mortar Materials

**Figure 13:**
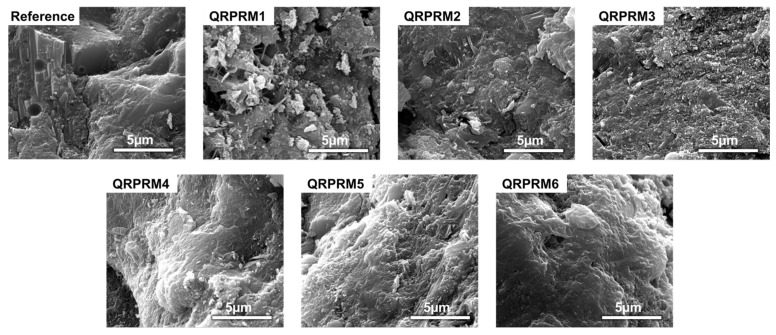
SEM images of VAE-RRM with different polymer-to-cement ratios.

Figure 13 shows the SEM images of VAE-RRM at different P/C values. From the images of the baseline group, it can be seen that the structure is relatively loose and the hydration products are relatively obvious. The network skeleton structure formed by the interconnection between hydrated calcium C-S-H, hexagonal calcium hydroxide (C-H), and the aggregate (Agg) is the main source of strength of cement-based materials and has good rigidity. However, due to the drying shrinkage of the matrix, microcracks and micropores appear between the structures, and the cement products are mostly connected to each other by weak van der Waals forces. So, the flexural strength and bonding strength performance are poor.

When the P/C is 0.3–0.9%, the microscopic morphology of the mortar changes significantly. With the increase in the amount of VAE powder, the hydration products are more and more closely connected to the polymer film, and the amorphous C-S-H gel is more completely generated around the cement particles and filled in the pores. It can be seen from the figure that there are no more tiny pores compared to in the baseline group, and the pores are filled with flexible structures which provide flexible support without affecting water penetration and improve the bending performance, which is the same as the conclusion of the mechanical properties experiment.

When the P/C is 1.2–2.0%, with the increase in polymer powder content, the film-like structure of the powder adheres to the surface of the hydration products, which greatly limits the formation and growth of hydration products such as AFt, C-S-H, and CH, occupies the position of the hydration products in the skeleton network structure, and affects the stiffness of the mortar structure; when the P/C is 2%, many tiny holes can be seen on the surface of the mortar. This is because the polymer film formed in the early stage has water retention and will lose water in the later stage of mortar hydration, resulting in the formation of tiny pores inside the polymer film [34]. Some gases are not discharged in time during the formation of the polymer film and remain in the mortar, eventually leading to the formation of harmful pores, which will cause the organic components to be loosely connected with the inorganic components in the cement, thereby adversely affecting the overall strength of the structure. Therefore, when the VAE powder content is excessive, the mechanical and bonding properties show a downward trend.

#### 3.1.9. Energy Dispersive Spectroscopy (EDS) Analysis of Content

**Figure 14:**
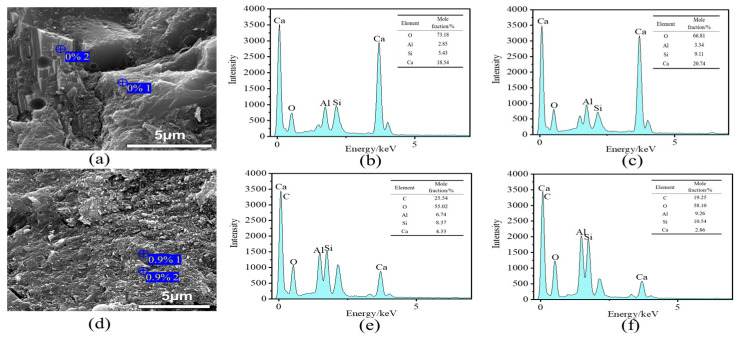
EDS analysis of VAE-RRM with different polymer-to-cement ratios. (**a**) SEM images of reference; (**b**) EDS analysis of Site 1 in reference; (**c**) EDS analysis of Site 2 in reference; (**d**) SEM images of VAE-RRM3; (**e**) EDS analysis of Site 1 in VAE-RRM3; (**f**) EDS analysis of Site 2 in VAE-RRM3.

The two groups for morphology comparison, the benchmark group and VAE-RRM 3, were selected, and EDS point scanning was performed. The results are shown in Figure 14 below. The scanning results of the benchmark group are shown in Figure 14b,c. It is found that the main elements at this point are O, Al, Si, and Ca, indicating that this area is mainly composed of hydration products and aggregate components of C-S-H and AFt; EDS scanning of VAE-RRM 3 found that in addition to the above elements, C appeared, and the number of C atoms accounted for about 20%, which is much higher than the carbon-oxygen ratio (C/O) of 0.1 in ordinary cement mortar, indicating that in addition to inorganic cement-based materials, there are also organic polymer components in this area. Combined with Figure 14 and the test macroscopic performance data, it can be concluded that when the P/C is 0.9%, an obvious organic–inorganic network structure can be observed, and some hydration products are wrapped by the polymer film and adhere to each other to form a high-density composite network structure, providing better flexural and bonding strength performance.

### 3.2. Discussion

**Figure 15:**
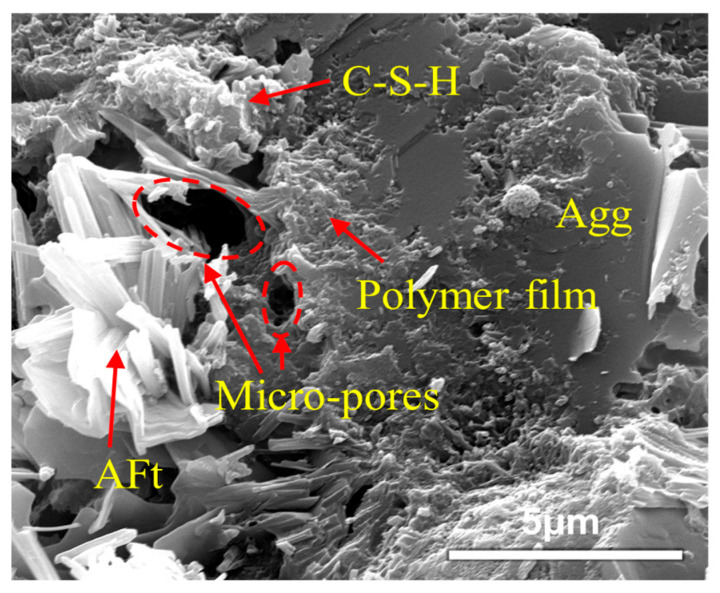
Three-dimensional network structure diagram of VAE-RRM.

**Figure 16:**
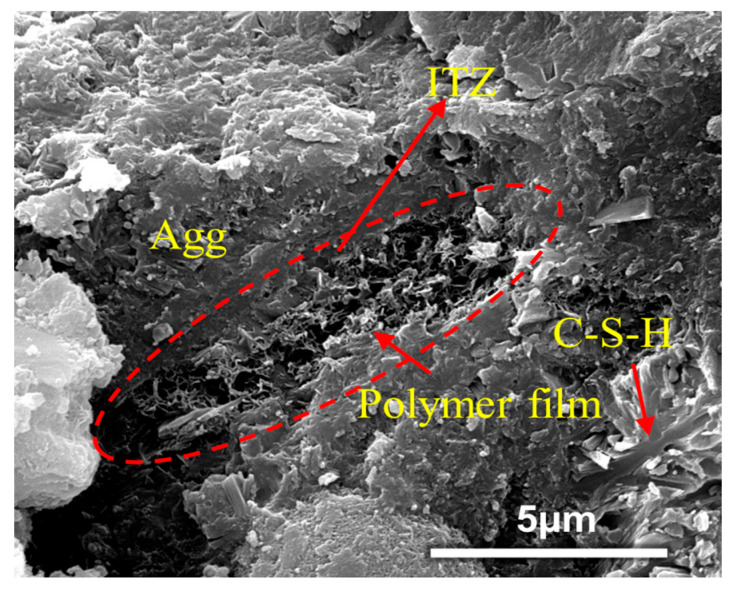
Polymer bridging effect diagram of VAE-RRM.

**Figure 17:**
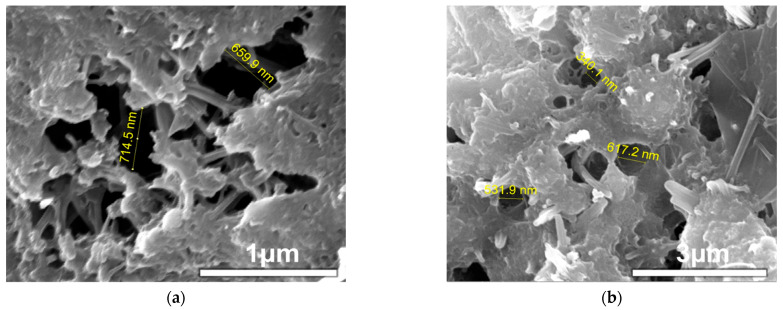
VAE-RRM pore distribution. (**a**) Reference; (**b**) VAE-RRM 3.

**Figure 18:**
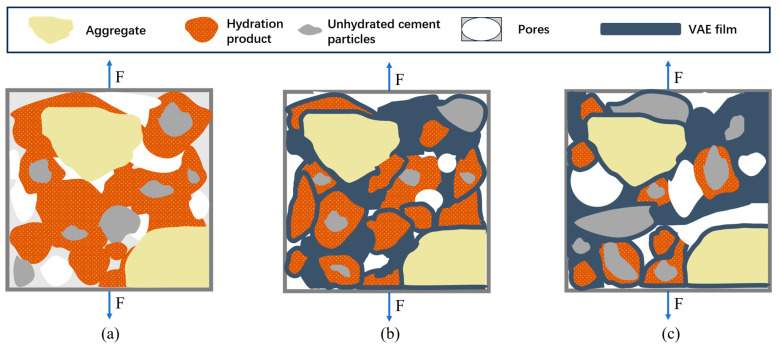
Load transfer and performance influence of VAE-RRM. (**a**) Reference mortar; (**b**) mortar modified with optimal amount of VAE; (**c**) mortar modified with excessive amount of VAE.

The modification mechanism of VAE polymer powder combined with OPC and SAC to form a composite cement-based material is analyzed at the microscopic level [35,36,37], and the main points are as follows:

1. Bridging effect. As observed in Figure 15, the incorporation of VAE powder promotes a bridging effect among hydration products in VAE-RRM, which aligns with the findings reported by Song et al. [38]. This consistency confirms the critical role of VAE in enhancing interfacial connectivity within the cementitious matrix. On the one hand, the polymer powder has high activity. After forming an emulsion with water, the active groups such as carboxyl in it will react with the free Ca^2+^, Mg^2+^, and Fe^2+^ ions in the cement hydration products to form a special bridge bond, which adsorbs the hydration products through chemical reactions to make the connection more dense. On the other hand, during the mixing process of the mortar with water, polymer particles disperse into the water to form a VAE emulsion. When the water evaporates and the curing temperature exceeds the minimum film-forming temperature of the polymer, the VAE emulsion particles lose water, come closer to each other, and form a colloidal film structure. This structure encapsulates the hydration products and fills some of the pores. As a flexible structure, the polymer film can produce a certain degree of deformation and has excellent bonding ability, connecting the hydration products of AFt, C-S-H, C-H and aggregates as well as cement particle components and improving the overall pore structure of the mortar to form a three-dimensional organic–inorganic network structure, thereby improving the performance of VAE-RRM.
2. Improving the interface transition zone (ITZ). As shown in Figure 16, the ITZ is located in the pore area between aggregates and cement hydration products. It is considered the weakest connection in cement-based materials and has a relatively important influence on the macroscopic mechanical properties and durability of the material. With the formation of the polymer film, the pores in the ITZ are filled, so that the contact between cement hydration products and aggregates is more compact, thereby improving the strength.
3. Optimizing the pore structure. As shown in Figure 17, in the benchmark group, the amorphous C-S-H gel and Aft grow around the cement particles, and the pores in the middle are connected with each other to form a 3D pore cluster with direct penetration between the hydration products. After adding the VAE polymer powder, the powder emulsifies and adheres to the surface of the hydration products, filling the original amorphous pores and forming an ellipse directly through the pores. The upper and lower layers of the staggered distribution form a sponge-like pore structure, and optimization of the pore structure not only enhances the overall seepage performance of the mortar but also optimizes its structural toughness.
4. Optimizing the Load Transfer Mechanism. When the mortar is subjected to external forces, the stresses should be transferred from the cement to the polymer through the interface. A schematic diagram of the load transfer mechanism at different polymer dosages is shown in Figure 18. When VAE is not added, the hydration products of the cement matrix are rigidly connected and cannot withstand the structural deformation brought about by load application, resulting in brittle damage of the cement mortar.

However, the addition of an appropriate amount of VAE can effectively improve the bonding and flexural strength. This may be related to the difference in the modulus of elasticity of the two materials, where the cement matrix has a modulus of elasticity of about 15–30 GPa and VAE has a modulus of elasticity of about 300 MPa. The polymer suffers more deformation when the load is applied to disperse the stresses. In addition, the continuous network formed by the polymer may help to share some of the load, especially when microcracks appear in the cement matrix, and VAE acts as a bridge to stop the cracks from expanding.

When VAE is added in excess, it not only inhibits the hydration reaction of cement, but it may also form microcracks or bubbles during water loss and shrinkage, introducing structural defects. In addition, after VAE exceeds a certain proportion, the original polymer as the reinforcing phase becomes a continuous phase, and the cement matrix also becomes a dispersed phase, so the performance of the whole material may more closely resemble that of the polymer, and the strength of the cement cannot be utilized.

## 4. Conclusions

1. Through the design of different VAE mixing amounts, for OPC and SAC binary compounding fast repair mortar, its performance under the influence of different conditions was investigated and analyzed. We found that with P/C enhancement, setting time was prolonged. In terms of mechanical properties, the compressive strength showed a downward trend, while flexural strength and adhesive strength showed a trend of increasing and then decreasing. When the P/C was 0.9%, the 28 d compressive strength and the compression–flexure ratio were 58 MPa and 4.49, a decrease of 10.2% and 50.1% compared with the benchmark group. The 28 d flexural strength and 28 d adhesive strength were 14.62 MPa and 2.74 MPa, an improvement of 34.0% and 182.5% compared with the benchmark group, and toughness was significantly improved.

2. The MIP method was selected to analyze the pore structure of VAE-RRM. It was found that when the P/C ratio increased, the porosity first decreased and then increased. The reason lies in that the addition of VAE closes and fills the harmful pores in the cement paste, and the polymer film separates large harmful pores into smaller transition pores. This reduces the number of large pores and capillaries, and the size of the largest pores become smaller transition pores, thereby densifying the mortar structure. Such pore size migration contributes to a denser mortar structure.

As the P/C ratio continued to increase, increased VAE doping had two effects: on the one hand, it produced an air-entraining effect on the overall mortar; on the other hand, water loss and crumpling of the glue powder membrane led to the formation of more pores. When VAE doping was too high, cement hydration was significantly affected, resulting in internal structural deterioration. At this point, the deteriorating effect of VAE exceeded the optimization effect on the pore structure, causing the overall porosity to increase.

It was also found that a certain amount of VAE powder could optimize pore size structure. The pore structure exhibited a multi-peak distribution, with the peak area concentrated in the 30 nm to 220 nm range. Pore dispersion was more uniform and continuous, which densified the mortar structure and enhanced the flexural and bonding properties.

3. Combined with secondary electron microscopic morphology (SEM), energy spectrum elemental analysis (EDS), and other multiple means to explore its comprehensive performance improvement mechanism, it was found that the addition of VAE could make each hydration product and polymer gel film connect closely; the amorphous C-S-H gel was generated more completely around the cement particles, the polymer gel film filled the inside of the pores, and the hydration products of the cement and polymer gel film connected with each other to fill in the pores and form a kind of denser three-dimensional skeleton structure that optimized the ITZ region of the material. However, through the SEM observations, it was found that VAE could improve the pore structure and enhance the toughness and strength of VAE-RRM by improving the shape and arrangement of pores. However, when the P/C was too high, the membrane structure of the gum powder restricted the formation and growth of hydration products and affected the hydration process. Finally, the incorporation of excessive VAE led to the formation of harmful pores, which adversely affected the overall strength of the structure.

## Figures and Tables

**Table 1:** Physical mechanical properties of experimental cementitious materials.

Index	Setting Time (min)		Flexural Strength (MPa)		Compressive Strength (MPa)		Specific Surface Area (m^2^/kg)	Stability (Boiling Method)
	Initial	Final	3 d	28 d	3 d	28 d		
P.O 42.5	175	239	6.0	5.9	31.9	50.9	363	Qualified
HBSAC 42.5	22	31	6.6	7.0	33.7	46.2	501	Qualified

**Table 2:** Chemical composition of experimental cementitious materials.

Index	CaO	SiO_2_	Al_2_O_3_	SO_3_	Fe_2_O_3_	MgO
P.O 42.5	52.09	23.47	8.55	2.43	4.11	3.26
HBSAC 42.5	40.67	18.33	16.93	14.17	1.51	5.71

**Table 3:** Basic properties of VAE powder.

Solids Content (%)	Ash Content (%)	Bulk Density (kg/m^3^)	Particle Size After Redispersion (µm)	Particle Size After Redispersion (µm)	Material Characteristics
99 ± 1	11 ± 2	390–420	0.5–8.0	4.0	Opaque film formation with high toughness

**Table 4:** Proportions of the mix for strengthening and repairing.

Group No.	(P/C)/%	Water	Cement	Sand	Polymer Powder	Water Reducing Agent
Reference	0	300	800	700	0	0.6
VAE-RRM 1	0.3				2.4	
VAE-RRM 2	0.6				4.8	
VAE-RRM 3	0.9				7.2	
VAE-RRM 4	1.2				9.6	
VAE-RRM 5	1.5				12.0	
VAE-RRM 6	2.0				16.0	

**Table 5:** Compression–flexure ratio of VAE-RRM.

Group No.	Compression–Flexure Ratio	
	7 d	28 d
Reference	6.9	7.3
VAE-RRM 1	6.3	6.7
VAE-RRM 2	5.1	5.6
VAE-RRM 3	4.6	4.5
VAE-RRM 4	4.1	3.9
VAE-RRM 5	4.0	3.5
VAE-RRM 6	4.1	4.1

**Table 6:** Mechanical properties of basic test blocks.

Test Project	7 d	28 d
Bending strength/MPa	10.1	10.3

## Data Availability

The original contributions presented in this study are included in the article. Further inquiries can be directed to the corresponding author.

## Abbreviations

VAE-RRM	Vinyl acetate-ethylene rapid repair mortar
VAE	Vinyl acetate-ethylene
PRM	Polymer-reinforced mortar
AC	Acrylic
SAE	Styrene-acrylic emulsion
SBR	Styrene-butadiene rubber
OPCPRM	Ordinary Portland cement polymer-reinforced mortar
SACPRM	Sulfoaluminate cement polymer-reinforced mortar
CPRM	Polymer repair and reinforcement mortar with a variety of cementitious materials in a compounding system
BTRM	Basalt fiber-reinforced polymer mortar
MIP	Mercury intrusion porosimetry
SEM	Scanning electron microscope
EDS	Energy dispersive spectroscopy
FTIR	Fourier-transform infrared spectroscopy
AFt	Ettringite
C-S-H	Calcium Portland hydrate
C-H	Calcium hydroxide
ITZ	Interfacial transition zone
P/C	Polymer/cement
W/C	Water/cement
C/S	Cement/sand
C/CR	Cement/cement water-reducing agent
